# Changes in Major Phenolic Compounds of Seeds, Skins, and Pulps from Various *Vitis* spp. and the Effect of Powdery and Downy Mildew Diseases on Their Levels in Grape Leaves

**DOI:** 10.3390/plants10122554

**Published:** 2021-11-23

**Authors:** Arif Atak, Zekiye Göksel, Yusuf Yılmaz

**Affiliations:** 1 Department of Viticulture, Atatürk Horticultural Central Research Institute, Yalova 77102, Turkey; 2 Department of Food Quality, Atatürk Horticultural Central Research Institute, Yalova 77102, Turkey; zekiyegoksel@gmail.com; 3 Department of Food Engineering, Faculty of Engineering and Architecture, Burdur Mehmet Akif Ersoy University, Burdur 15030, Turkey; yilmaz4yusuf@yahoo.com

**Keywords:** gallic acid, catechin, epicatechin, fungal diseases, grape berry, hybrids

## Abstract

The main purpose of this study is to determine the contents of 3 major phenolic compounds (gallic acid, catechin, and epicatechin) in 22 different grape cultivars/hybrids obtained from 2 different breeding programs. Additionally, changes in these phenolic components in the grape leaves of some resistant/tolerant species were determined in relation to powdery and downy mildew diseases in viticulture. The skin, pulp, and seeds of grape berries were analysed over two years, while changes in the phenolic contents of grape leaves were determined before and after these diseases for two years. The major phenolic contents of new hybrids/cultivars were compared with those of popular cultivars in different parts of the grapes, and significant differences in phenolic contents were found among hybrids/cultivars and different grape parts. Variations in the contents of phenolics in grape seeds, skins, and pulp were high, but seeds contained higher levels of these phenolics than pulp and skin. Analyses of the relationship between two viticultural diseases and phenolic changes in resistant/tolerant cultivars in relation with the susceptible “Italia” cultivar revealed that an increase in the content of the phenolic compounds was found after powdery mildew disease. Hybrids/cultivars with high phenolic contents are recommended to develop new superior cultivars, which are resistant to grape fungal diseases, in breeding programs.

## 1. Introduction

Grapes are one of the richest sources of phenolics among fruits, and many of them are renowned for their therapeutic or health-promoting properties, making grapes an important fruit for human health [1]. Phenolic compounds are an extensive family of numerous natural bioactive compounds with health benefits [2] and constitute one of the most common and widespread groups of substances in plants [3]. Most of the phenolic compounds in plants are important for pigmentation, plant reproduction activities, juice and wine production and flavour formation, and as substrates for enzymatic browning, while playing an important role in the resistance of plants against diseases [4]. The concentration of these compounds in different parts of plants may vary, and stress factors, to which plants are exposed, may change their contents in grape berries [5]. Although the phenolic profiles of grapes depend on various factors, such as cultivar, maturity [6], genetic diversity [7], viticulture practices [8], soil characteristics [9], environmental stress and vine health status [9], the composition of phenolic compounds in grape berries strongly depends on cultivars [10,11,12]. The distribution of phenolic compounds in grape berries seems irregular. About 64% of the total of free phenolic compounds are found in seeds, 30% in skins, and 6% in pulps, and phenolic compounds in seeds, skin, and pulp are represented by flavan-3-ols, flavonols, and hydroxycinnamic acids, respectively [13,14]. Grape seeds and skins are important sources of phytochemicals, such as gallic acid, catechin, and epicatechin, and suitable raw materials for the production of antioxidative dietary supplements [15,16].

Secondary metabolites in plants may act as a part of defence mechanism against herbivores, microbes, viruses, and competing plants, and signal compounds to attract pollinating or seed-dispersing animals, as well as protection of the plant from ultraviolet radiation and oxidants [17,18]. While plant phenolics as secondary metabolites may inhibit the growth of insects [19], their resistance role against fungi is more dynamic than their role against insects. Infection, wounding or herbivory, in plants may induce the production of several classes of secondary metabolites. Genetic variation in the speed and extent of such induction may account for the difference between resistant and susceptible cultivars. When plants are exposed to diseases, a rapid accumulation of phenolic compounds are usually observed as a part of their defence mechanism in the infected part first, and this slows down the development of pathogens [20]. Researchers working on this topic have previously found a correlation between increased host resistance and high phenolic compound content [21,22]. A variety of factors contribute to the ability of plants to resist attacks by different pathogenic microorganisms, and the level of phenolics in different parts of grapes may differ. Concentration of phenolics may increase significantly after the infection of fungal diseases [23,24].

Downy mildew (*P. viticola*) and powdery mildew (*U. necator*) are the most common destructive grapevine diseases that occur worldwide, particularly in warm and humid climates [25]. According to recent global surveys, researchers and agricultural professionals in the main grape growing regions have considered these diseases as the most harmful for grape production [26,27]. Although these diseases damage almost all the above-ground organs of vine, they cause great damage, especially in grape berries. With different breeding studies carried out in the world, new grape cultivars resistant to these diseases are developed continually [28,29]. Such studies are carried out by two different exclusive research institutes, Tekirdağ Viticulture Research Institute (TVRI) and Yalova Atatürk Horticultural Central Research Institutes (YAHCRI), in Turkey for many years [29]. The cultivars and hybrids used in this study have been developed under the breeding programs of these institutes.

Flavonoids are a group of the most abundant biologically active phytonutrients among polyphenolic compounds present in grapes, and they represent a large family of secondary metabolites, with nearly 6000 structures identified in plants. Catechin and epicatechin are among the most common flavonoids found in grapes [30,31]. Gallic acid is a trihydroxybenzoic acid and classified as a phenolic acid, which may have various therapeutic properties, including antioxidant, anti-cancer, anti-inflammatory, antifungal, and antiviral activities [32]. Gallic acid, catechin, and epicatechin have been studied by many researchers, especially their concentrations in different parts of grape berries from various cultivars because of their health-beneficial properties [33,34].

Monitoring the major phenolics of grape berries and response of grape plants to viticultural diseases are critical for the selection of superior cultivars/hybrids in the breeding programs of viticultural studies. Therefore, the contents of gallic acid, epicatechin, and catechin in three different berry parts (pulp, skin, and seed) of grape cultivars/hybrids from the breeding programs of TVRI and YAHCRI were monitored for two years in the first part of the study. In the second part, cultivars/hybrids that were resistant/tolerant to powdery and downy mildew diseases at different rates were selected and the contents of gallic acid, epicatechin, and catechin in grape leaves were evaluated before and after the disease. Finally, the relationship between the contents of phenolics and the diseases of cultivars/hybrids were determined.

## 2. Materials and Methods

### 2.1. Plant Materials

In the first part of the study, the contents of 3 different phenolic compounds present in 3 different parts of grape berries (skin, pulp, and seed) were determined in 22 cultivars/hybrids grafted on Kober 5 BB rootstocks over two years. The properties of grape cultivars/hybrids used in this first part are given in Table 1.

Standard grape cultivars were grown in the Marmara region, northwest Turkey, with an exception of Isabella, a local grapevine cultivar in the Black Sea region in northern Turkey. It is grown especially in humid areas because of its resistance to fungal diseases. Other cultivars/hybrids were selected from the disease-resistant cultivation breeding program of TVRI (Tekirdağ, Turkey). The fresh berry samples were harvested from 22 different cultivars between the third week of August and end of September during two consecutive growing seasons. Soluble solid levels ranged from 25.3 to 30.4° Brix during the harvest. Plants were grown at the ACHRI vineyards in Yalova, Turkey.

In the second part of the study, 10 grape cultivars/hybrids were inoculated with powdery and downy mildew diseases artificially in a greenhouse over 2 years. *V. vinifera* “Italia” was used as control because of its sensitivity to the diseases. Related analyses were conducted in the Food Technology Laboratory at YAHCRI. Disease inoculations were carried out in a greenhouse using 2-year-old potted vines grown in 5 L pots filled with a soil mixture (1/3 garden soil, 1/3 peat moss, and 1/3 compost). Optimum climatic conditions were ensured for the development of both diseases in the greenhouse.

### 2.2. Inoculation of Vines and Evaluation of Fungal Diseases

All cultivars/hybrids were planted in pots and cultivated in the greenhouse for downy and powdery mildew inoculations. Inoculation was applied according to Wang et al. [35] and Boso et al. [36]. Fungal conidia were collected from infected leaves from the YAHCRI vineyards. For the propagation of powdery mildew, inoculum vines were sprayed with a suspension of sporangia (40,000 sporangia per mL of distilled water) on the abaxial leaf side and plants were completely covered with polyethylene covers overnight. Next day, polyethylene covers were removed, and incubation lasted 5–6 days at 25 °C. This procedure was repeated after a week.

The vine leaves were inoculated with a conidial suspension at 2 × 10^5^ conidia mL^−1^ by spraying the upper surface of the leaves for downy mildew inoculation. The inoculated leaves were immediately covered with thin plastic for 6 h. Fogging was applied for a limited period in order to stimulate the formation of the diseases at a desired level. Both disease inoculations were taken place independently in two separate compartments in the greenhouse.

Depending on the vigour of vines, four-to-six young leaves from the shoot tip were selected from each vine, and were observed for powdery mildew at different times during June–August. The severity of infections on leaves was determined based on the percentage of disease spots observed on the entire leaf area [37], according to the procedure described in Table 2. Disease severity was scored 3 weeks after inoculation. Since plants in a greenhouse usually develop faster than those in an open field, disease inoculation and scoring were done earlier. All leaves of each plant were observed at different times during May–August for a downy mildew disease. The infection severity on leaves was determined based on the percentage of disease spots observed on the entire leaf area (Table 2). Scoring was done after 6 weeks of inoculation.

### 2.3. Collection and Preparation of Samples

Samples in the first part of the study were taken as follows: the clusters of cultivar/hybrids were checked every week for the ideal harvest time, and those that reached the ideal Brix ratio were harvested. At the time of harvest, 2–3 kg samples were collected from different vines and different parts of vines, representing each cultivar. A bunch of grapes was brought to the laboratory immediately after harvest. The seed, skin, and pulp parts of the grapes were carefully separated manually. Each part was divided into three equal parts, representing replicates. Seeds were partially dried at 45 °C for 4 h in a convection oven (Memmert UN110, Nurnberg, Germany) in order to facilitate the grinding process. The pulp and skin parts were frozen at −20 °C until analysis. The partially dried seeds were ground with a coffee grinder (Bosch, MKM 6000, Istanbul, Turkey), whereas the pulp or skin parts were chopped with a blender immediately after thawing. Analyses were performed in duplicates within a month.

Grape cultivars/hybrids in the second part of the study were kept in separate sections in the greenhouse with 3 replications and at least 3 vines in each repeat. Samples were collected from all replicates before and after diseases. The first 6 leaves of the grape cultivars/hybrids from the shoot tip (before and 3 weeks after disease inoculation) were used for analyses. The first 6 leaves of each of the 3 vines (healthy and infected vines) were used, and the leaves were separated carefully from their petioles and washed in pure water. Each part was divided into three equal parts, representing replicates. Clean leaves were dried for 48 h at room temperature under dark. Dried leaves were ground at a high speed for 60 s by a grinder, and then 2 g of leaf powder was added to 40 mL of methanol and shaken for 60 min at 60 °C in a water bath. The samples were centrifuged for 10 min at 7000 rpm (5810 Eppendorf AG, Hamburg, Germany). The supernatants were collected in amber bottles and stored at −20 °C until analysis. Three extracts were obtained for each grape cultivar/hybrid: the first week of June (healthy), end of June (downy mildew), and end of July (powdery mildew).

### 2.4. Determination of Major Phenolic Compounds

The contents of gallic acid, catechin, and epicatechin in the leaf extracts of grape cultivars/hybrids were determined by using a chromatographic unit (HP1100 System HPLC, Agilent Technologies Inc., Palo Alto, CA, USA), according to the method of Katalinic et al. [38] with some modifications.

The separations were conducted at room temperature in an Agilent Eclipse XDB-C18 column (4.6 × 250 mm, particle size 5 µm), protected by a guard column. The compounds were detected with an HP 1100 series ultraviolet (UV) Diode Array Detector (DAD). The mobile phase included A: 2.0% acetic acid in distilled water, and B: acetonitrile. The column was eluted at 1.0 mL/min under a linear gradient from 5% mobile phase B to 75% over 20 min, to 100% over 5 min, isocratic for 5 min, to 25% over 5 min, and to 5% over 5 min. The injection volume was 20 µL for each sample. Phenolic compounds were identified according to the retention times of the available pure compounds and the UV–Vis data obtained from authentic standards and/or published in previous studies [39]. Gallic acid, catechin, and epicatechin (Sigma-Aldrich) were quantified at 280 nm. Their contents in grape seeds, skins, and pulps and leaf samples were expressed as milligrams per weight (mg 100 g^−1^). The results of these phenolic compounds were the averages of triplicates.

### 2.5. Statistical Analysis

An analysis of variance (ANOVA) was used to determine significant differences among means. The data were presented as arithmetic means of three replications ± standard deviations, which represented the means of two consecutive years. For each parameter, the LSD (the least significant difference) was used to determine the level of significant differences for all accessions. Differences at *p* ˂ 0.05 were considered significant. The correlation coefficients (R) of a parametric Pearson’s test was used to evaluate covariance relationships among variables. The statistical analyses were performed using JMP 15.0 software [40].

## 3. Results

### 3.1. Evaluation of Fungal Diseases

The resistance level of the cultivars/hybrids against downy and powdery mildew diseases was monitored for two years after the artificial inoculation of vine, and results are given in Table 3. Results indicated that the cultivar “Isabella” was very resistant to downy mildew disease, while the cultivar “Özer Karası” and the hybrid of FX1-1 exhibited resistance to this disease (with a score of 3). Other five cultivars/hybrids determined as tolerant to downy mildew diseases.

In terms of a powdery mildew disease, results, which are similar to the scores for downy mildew diseases, were obtained in general (Table 3). The cultivar “Italia”, known to be susceptible to powdery mildew diseases, was used as a control cultivar, and it was found “susceptible” to powdery mildew diseases in our study. In addition, the 86/1 hybrid had a score of 7, indicating that it was “susceptible” to powdery mildew diseases. Of the other cultivars/hybrids, “Isabella” and “FX1-10” had “a high resistance”, while “FX1-1” and “Özer Karası” were resistant. “Güzgülü”, “BX1-166”, and “KXP-10” showed “tolerance” to powdery mildew disease.

### 3.2. Determination of Phenolic Compounds in Grape Leaves

The gallic acid, catechin, and epicatechin contents of grape leaves from different cultivars/hybrids were monitored for two years before and after downy and powdery mildew diseases, and results are given in Table 4. In addition, Pearson’s correlation coefficients among the contents of phenolic compounds in grape leaves before and after diseases are given in Table 5, representing the averages of the two harvesting seasons.

The catechin contents of grape leaves for different grape cultivars/hybrids were compared in Table 4 within each harvesting season, and they seemed to fluctuate over the two harvesting seasons, although a statistical comparison was not included in the study. In the first year, the catechin contents remained low during the healthy period of the first harvesting season before mildew diseases; they increased especially after powdery mildew infections. It was noticeable that the catechin contents of leaves before diseases in the second year were generally higher than those in the first year. The values in Table 4 indicate that the effect of diseases on the catechin contents of leaves was dependent on the grape cultivar/hybrid. In terms of the numerical results in Table 4, although the catechin content of grapes leaves for the “Italia” cultivar, highly susceptible to diseases, increased after diseases, the leaves of the disease-resistant cultivars “Isabella” and “Özer Karası” had a low content of catechin in general. These results show that the catechin content in grape leaves may be influenced by mildew diseases differently depending on the susceptibility of grape vine to diseases. Among all cultivars/hybrids, the grape leaves of “KXP-10” and “Özer Karası” (disease resistant) with dark berries had a catechin content of about 54 mg 100 g^−1^ in the healthy period before diseases (Table 4). In terms of correlations within catechin itself, the highest, but insignificant correlation (0.49) was found between the values obtained before disease (Catechin, BD) and after powdery mildew disease (Catechin, PM) (*p* > 0.05).

The epicatechin contents of grape leaves increased numerically for almost all cultivars/hybrids in the 1st year of harvesting, regardless of mildew disease conditions in plants. In the 2nd year, this trend was unclear. Although seasonal changes in phenolics or the effect of diseases on major phenolics were not compared statistically in this study, this result could be attributed to the fact that the epicatechin response of vine might be highly influenced by the harvesting season (most likely climatic conditions in a season) and the severity of mildew diseases as well as the variety of grapes. An increase in the epicatechin contents was observed for the leaves of the “Italia” cultivar, which is more susceptible to mildew diseases than others, after diseases. The epicatechin contents of grape leaves from the disease-resistant cultivars “Isabella” and “Özer Karası” seemed to increase especially after powdery mildew disease. In terms of the averages of the two harvesting years, the catechin content of grape leaves increased for the cultivars/hybrids, which was much more noticeable after powdery mildew. Among the grape cultivars/hybrids studied, the epicatechin content of the “86/1” hybrid with yellow flesh colour and intense Muscat flavour after powdery mildew infection in the 1st year of harvesting was 427 mg 100 g^−1^. After downy mildew infection, the leaves of the “KXP-10” hybrid in dark fruit colour had an epicatechin content of about 87 mg 100 g^−1^.

The results of the correlation study within epicatechin itself show that the highest, but insignificant correlation (R = −0.40) was found before diseases (Epicatechin, BD) and after powdery mildew disease (Epicatechin, PM) (*p* > 0.05). When we look at the correlation between other phenolic compounds and epicatechin, the epicatechin content of grape leaves after downy mildew disease (Epicatechin, DM) was significantly and positively correlated with the catechin content of leaves after powdery mildew (Catechin, PM) (R = 0.71), but negatively correlated with the gallic acid content before disease (Gallic acid, BD) (R = −0.70) (*p* < 0.05). Moreover, the epicatechin content of grape leaves after powdery mildew disease (Epicatechin, PM) was positively correlated with the gallic acid content after powdery mildew disease (Gallic acid, PM) (R = 0.69) (*p* < 0.05).

In general, small increases in the gallic acid contents of grape leaves were found numerically in the 2nd harvesting year after powdery mildew infection for all cultivars/hybrids. Furthermore, the gallic acid contents of grape leaves seemed to decrease after downy mildew infections in comparison to the healthy period. The reduction in the gallic acid contents of grape leaves before the disease and after downy mildew infection in the 2nd harvesting year was similar for all cultivars/hybrids. Among all cultivars/hybrids studied, the gallic acid contents of grape leaves after powdery mildew disease were 6.51 mg 100 g^−1^ for the “86/1” hybrid with yellow berry colour and intense Muscat flavour and 4.98 mg 100 g^−1^ for the “KXP-10” hybrid with dark berry colour in the 2nd year of harvesting. Especially after powdery mildew, the grape leaves from the cultivars/hybrids with intense Muscat flavour and dark coloured berries tended to have higher gallic acid contents than those with less flavoured and light coloured berries.

The highest, but insignificant correlation (R = −0.37) was found between the gallic acid contents of grapes leaves before disease values (Gallic acid, BD) and downy mildew infection (Gallic acid, DM) (*p* > 0.05). Negative and significant correlation coefficients in Table 5 indicate that the gallic acid contents of healthy vines decreases after powdery mildew infection or vice versa. As reported in the previous paragraph, the gallic acid content of grape leaves after powdery mildew disease (Gallic acid, PM) was positively correlated with the epicatechin content after powdery mildew disease (Epicatechin, PM) with R = 0.69 (*p* < 0.05).

### 3.3. Determination of Phenolic Compounds in Different Parts of the Grape Berry

The contents of catechin, epicatechin, and gallic acid in the skins, pulps, and seeds of grape berries from 22 cultivars/hybrids for two years are given in Table 6, Table 7 and Table 8.

Especially the seed parts of grape berries from the cultivars/hybrids studied contained generally high amounts of catechin in comparison to their skin or pulp parts. The catechin contents of grape pulps ranged from 0.50 to 0.81 mg 100 g^−1^ in the 1st year and from 0.54 to 0.76 mg 100 g^−1^ in the 2nd year, while the catechin contents of grape skins ranged from 0.03 to 4.59 mg 100 g^−1^ in the 1st year and from 0.03 to 3.22 mg 100 g^−1^ in the 2nd year. Moreover, grape seeds had a catechin content ranging from 48.17 to 494.91 mg 100 g^−1^ in the 1st year and from 44.95 to 494.95 mg 100 g^−1^ in the 2nd year. The highest catechin content in seeds was detected for the “KXP-10” hybrid grapes as 492 mg 100 g^−1^ in the 1st year and 495 mg 100 g^−1^ in the 2nd year of harvesting (*p* < 0.05). Additionally, the second highest catechin content was determined for the seeds of the “85/1” hybrid, which has the yellow coloured berries and intense Muscat flavour (about 370 mg 100 g^−1^) (*p* < 0.05). The pulp and skin parts of the grape cultivars/hybrids studied had a low content of catechin. In terms of the catechin content of skins and pulps, a high content was determined in cultivars/hybrids with dark coloured berry, such as “Isabella” and “Trakya İlkeren”, or light coloured berries, such as “85/1” and “86/1”, containing an intense Muscat flavour. Catechin could not be detected in the skins of two cultivars/hybrids (“Güz Gülü” and “130/1”) in both years, probably due to its very low content (Table 6).

The seeds of the grape cultivars/hybrids studied contained more epicatechin than their skins or pulps for the two harvesting years. The epicatechin contents of grape pulps ranged from 0.01 to 1.06 mg 100 g^−1^ in the 1st year and from 0.01 to 1.23 mg 100 g^−1^ in the 2nd year. The epicatechin contents of grape skins ranged from 0.14 to 1.79 mg 100 g^−1^ in the 1st year and from 0.10 to 1.79 mg 100 g^−1^ in the 2nd year. Morevoer, the epicatechin contents of the grape seeds ranged from 20.65 to 106.91 mg 100 g^−1^ in the 1st year and from 20.50 to 107.20 mg 100 g^−1^ in the 2nd year. Among the cultivars/hybrids, the highest epicatechin content was determined in the seeds of “86/1” hybrid grapes with yellow-coloured fruits and an intense Muscat flavour (107 mg 100 g^−1^), which was followed by the seeds of the “KXP-10” hybrid grapes with black berry colour in both years (about 100 mg 100 g^−1^) (*p* < 0.05). The epicatechin content of the seeds of “Isabella” and “Özer Karası”, two dark berry cultivars, was about 21 mg 100 g^−1^, and their epicatechin content was the lowest (*p* < 0.05). The high levels of epicatechin in pulps and skins were obtained from cultivars with dark berry colour, such as the “Bilecik İrikarası” and “İsmetbey” cultivars. The skins or pulps of the cultivar “Pembe 77” grapes with a dark pink berry colour had a relatively high epicatechin content. The epicatechin contents in the pulps or skins of some grape cultivars/hybrids could not be detected in some of the harvesting years, possibly due to the existence of their very low levels (Table 7).

Based on the results of grape pulps, the gallic acid contents ranged from 0.57 to 0.76 in the 1st year and from 0.56 to 0.76 mg 100 g^−1^in the 2nd year. The gallic acid contents of the grape skins ranged from 0.59 to 1.47 mg 100 g^−1^in the 1st year and from 0.58 to 1.43 mg 100 g^−1^in the 2nd year. Moreover, the gallic acid contents of the grape seeds ranged from 1.35 to 6.88 mg 100 g^−1^in the 1st year and from 1.26 to 6.88 mg 100 g^−1^ in the 2nd year. Among different grape parts, grape seeds had the highest content of gallic acid in the manner of the other two phenolic compounds. Furthermore, compared to the catechin and epicatechin levels, the content of gallic acid in seeds was somewhat limited. The seeds of the “İsmetbey” cultivar grapes with black berry colour contained the highest gallic acid content (about 6.9 mg 100 g^−1^) in the 1st and 2nd harvesting years (*p* < 0.05) while its difference from the seeds of “FX1-10” was found insignificant in the 2nd year (*p* > 0.05). Moreover, in the 1st year, the second highest gallic acid content was determined in the seeds of the “FX1-10” hybrid grapes with yellow berry flesh colour. In the pulps and skins, the gallic acid contents were generally found high for the cultivars/hybrids with dark berry colour, such as “Isabella”, “83/1”, “Pembe 77”, and “Reçel Üzümü”, and for the hybrid of “86/1” with an intense Muscat flavour (Table 8).

Correlations among major phenolic components in three different parts of the grape berries were determined over the averages for two harvesting seasons. A positive and highly significant correlation coefficient (R = 0.95) was found between the catechin content of pulps (Catechin, Pulp) and the gallic acid of skins (Gallic acid, Skin) (*p* < 0.05). In addition, significant correlations were found for the phenolic components of grape seeds. While the correlation between gallic acid (Gallic acid, Seed) and the epicatechin contents of seeds (Epicatechin, Seed) (R = 0.77) was high and significant (*p* < 0.05), the catechin content of seeds (Catechin, Seed) was positively correlated with the epicatechin contents of seeds (Epicatechin, Seed) (R = 0.74) (*p* < 0.05). Moreover, there was a positive correlation coefficient between the catechin (Catechin, Seed) and gallic acid contents of seeds (Gallic acid, Seed) (R = 0.70) (*p* < 0.05). This correlation analysis showed that the contents of almost all phenolic compounds in grape seeds increased more than those in the other two parts of grape berries (Table 9).

## 4. Discussion

In this study, changes in the catechin, epicatechin, and gallic acid contents of grape leaves after two important fungal diseases and differences in the contents of these compounds in skins, pulps, and skins of grape berries for two harvesting seasons were determined. After downy mildew and powdery mildew diseases, differences in the contents of these phenolic compounds were observed among grape leaves depending on the cultivar/hybrid, harvesting year, and the type of phenolic compounds.

Although there may be a variety of factors influencing the concentration of phenolic compounds in grapes, their concentration is highly dependent on the cultivar types and even species. There may be differences in the phenolic contents of grapes among different cultivars, as well as within the fruits of the same cultivar grown in different regions [41] Several factors, such as the type of cultivar, processing techniques, viticultural practices, geographical region, and climatic conditions, may significantly influence the phenolic compositions of grapes [42,43,44,45,46]. In a study on the contents of some phenolic compounds in different grape cultivars, Eyduran et al. [47] reported that the quantity of phenolic compounds could vary depending on the type of cultivars. Doshi et al. [48] also reported that the concentrations of rutin and quercetin hydrates representing the flavanols group of phenolics might change in different organs of grapevine, and sometimes it could be barely detected.

In species, such as *Vitis labrusca*, *Vitis riparia*, and *Vitis rupestris* hybrids, differences in the chemical structures of phenolic components have been reported in the literature. The contents of phenolic compounds may differ in *V. vinifera* species [49], and for this reason, in most hybrid cultivars that are tolerant or resistant to grapevine diseases, the composition of these components can flactuate considerably. In our study, it was noteworthy that the contents of some phenolic components were higher in the disease-resistant varieties, such as “Özer Karası” and “Isabella”, which are cross-bred between species, especially in their skins and pulps.

Yaman et al. [50] reported that resveratrol levels in two different grape cultivars might be dependent on the vegetation time, cultivar, and region. In another study on changes in the total phenolic contents and seven phenolic compounds (gallic acid, catechin, catechol, chlorogenic acid, o-coumaric acid, rutin, and quercetin) of the shoot tips from “Cardinal” and “Uslu” grape cultivars collected in different months, Baydar [51] reported that the concentration of these phenolic compounds varied depending on the type of cultivar and harvesting month. The flavonol and anthocyanin contents of five red fungus-resistant grape cultivars (“Frontenac”, “Maréchal Foch”, “Marquette”, “Sabrevois”, and “St. Croix”) were characterised from berry (skin, seed, and free-run must) to wine to evaluate varietal differences and relationships between the berry and wine composition by Gagne et al. [52], and they reported that the principal component analysis of berry composition showed significant differences among the cultivars. In our study, we also found that the contents of phenolic compounds varied depending on the type of grape cultivar/hybrid.

In the present study, there were significant increases in the contents of catechin, epicatechin, and gallic acid in 22 different cultivars/hybrids, especially after powdery mildew disease. Using two wine grape cultivars (“Cabernet Sauvignon” and “Sauvignon Blanc”) and a table grape (“Thompson Seedless”), Taware et al. [53] determined the total phenolic contents and some phenolic compounds in the leaves, berries, and wines from healthy and powdery mildew-infected grapes, and they reported higher phenolic contents in the leaves of wine grapes compared to “Thompson Seedless”. Moreover, they reported that this disease significantly altered the phenolic profile of the leaves, berries, and wines while the foliar infection resulted in the accumulation of phenolic compounds in leaves and reduction in berries and wines because of cluster disease infection. In a study by Romero-Perez et al. [54], concentrations of phenolic compounds increased considerably in grape berries infected by powdery mildew disease in comparison to healthy grape berries. Santos et al. [55] compared the contents of phenolic compounds and trans-resveratrol in different berry parts from *V. vinifera* and *V. labrusca* cultivars/genotypes, and reported that *V. labrusca* cultivars/genotypes contained more phenolic compounds and trans-resveratrol than *V. vinifera* cultivars. Dani et al. [56] found a high level of phenolic compounds in the leaves of a *V. labrusca* cultivar, and reported that these compounds reduced the vine damage from lipids and proteins significantly. This result could explain how and why *V. labrusca* or interspecies cultivars with high contents of phenolic compounds in our study had significantly minimum damages after the inoculation of diseases. Rebello et al. [57] determined the contents of some phenolic components in the skins, pulps, and seeds of the “BRS Violeta” cultivar, a hybrid grape, and reported a very high content of phenolic components in skins, most probably because of the very thick skin of this cultivar (46% of grape weight).

Coklar [58] investigated the phenolic profile of whole berry, skin, and seeds of the local “Ekşikara” (*V. vinifera*) cultivar and determined the effect of harvesting year and altitude of the vineyard location. Anthocyanins, resveratrol, rutin, and isorhamnetin-3-glucoside were mainly found in skins, while monomers and dimers of flavan-3-ols were detected mainly in seeds. In the study, altitude had a drastic effect on phenolic compounds in whole berry, skins, and seeds, and very high amounts of catechin and epicatechin, especially in the seeds. The quantities of gallic acid, epicatechin, and catechin varied depending on harvesting year. Our results were in good agreement with this study.

Based on the chemical structure, phenolic compounds are mostly classified into flavonoid and non-flavonoids. Flavonoids are found mainly in grape seeds and skins while proanthocyanidins in grapes are present mainly in berry skins and seeds. Grape seed proanthocyanidins comprise only (+)-catechin, (−)-epicatechin, and procyanidins, whereas grape skin proanthocyanidins comprise both prodelphinidins and procyanidins [16,59]. Procyanidins are dimers resulting from the union of monomeric units of flavanols (+)-catechin. Among grape cultivars, there are differences in procyanidin concentrations, but their profile remains mostly unchanged. Prodelphinidins are only present in grape skin and their monomers are catechin, epicatechin, gallocatechin, and epigallocatechin units. Proanthocyanidins (procyanidins and prodelphinidins) are the major phenolic compounds in grape seeds and skins, and about 60–70% of total polyphenols are stored in seeds [16,59]. Similarly, in our study, monomeric phenolic compounds, such as catechin and epicatechin, were found at a very high concentration in grape seeds in comparison to grape skins.

Mulero et al. [60] reported that the skin, pulp, and seeds of grapes contain an enormous amount of different phenolic compounds, while Rodriguez-Montealegre et al. [61] found that the phenolic composition of grapes and different grape parts may depend on multiple factors, including climate, ripeness, berry size, grapevine cultivar, and viticulture practices. Our results also indicated significant differences in the contents of phenolic compounds in different berry parts of grapes from various cultivars/hybrids. Moreover, the catechin and epicatechin contents of seeds were much higher than those of pulps and skins. Most phenolic compounds are located in different parts of grape berries, and it would be beneficial to re-evaluate the processing techniques for many food products so that the phenolic components migrate or are incorporated into these products. Many phenolic components originating from grape skins and seeds could be health-beneficial especially during processing for food products. Phenolic compounds are related to not only human health, but also the fight of plants against diseases, and their composition may vary under the effect of different stress factors for plants.

## 5. Conclusions

The contents of major phenolic compounds, such as catechin, epicatechin, and gallic acid, in grape leaves increased especially after powdery mildew disease, but this increase seemed to be independent from the cultivar being disease resistant/tolerant. In order to understand the phenolic response of vine plants against downy and powdery mildew diseases, more comprehensive studies are needed. In addition, registration studies for novel grape cultivars should be accelerated so that candidate grape hybrids/cultivars, such as “86/1”, “85/1”, and “KXP-10”, which contained high levels of major phenolic compounds, could be used in the grape juice industry. Besides these hybrids, (interspecies) cultivars, such as “Isabella” and “Özer Karası”, with a high content of phenolics could also be used as parents in future breeding studies planning to develop new grape cultivars with a rich phenolic content.

## Figures and Tables

**Table 1:** Main characteristics and origin of grape cultivars/hybrids used in this study.

Cultivar/Hybrid	Species	Origin of Material	Berry Colour	Special Flavour	Seed Status
Isabella	Interspecies *(V. labrusca* × *V. vinifera)*	Common Cultivar	Black	Foxy	Seeded
Özer Karası		TVRI *	Black	No	Seeded
BX1-166		TVRI	Yellow/Green	No	Seeded
FX1-1		TVRI	Yellow/Green	No	Seeded
FX1-10		YAHCRI	Yellow/Green	No	Seeded
Alphonse Lavallée	*V. vinifera*	Common Cultivar	Black	No	Seeded
Muscat Hamburg		Common Cultivar	Black	Muscat	Seeded
Yalova Misketi		YAHCRI **	Black	Muscat	Seeded
Trakya İlkeren		TVRI	Blue/Black	No	Seeded
Bilecik İrikarası		Common Cultivar	Black	No	Seeded
İsmetbey		YAHCRI	Black	No	Seeded
KXP-10		TVRI	Black	No	Seeded
Tekirdağ Çekdsz.		TVRI	Dark Red/Purple	No	Seedless
Reçel Üzümü		TVRI	Red/Black	No	Seedless
Güz Gülü		TVRI	Rose	No	Seedless
Pembe 77		YAHCRI	Dark Pink	No	Seeded
Uslu		YAHCRI	Rose	No	Seeded
83/1		YAHCRI	Rose	No	Seeded
85/1		YAHCRI	Yellow/Green	Muscat	Seeded
53/1		YAHCRI	Yellow/Green	No	Seeded
86/1		YAHCRI	Yellow/Green	Muscat	Seeded
130/1		YAHCRI	Yellow/Green	No	Seedless
Italia		Common Cultivar	Yellow/Green	Muscat	Seeded

* TVRI, Tekirdağ Viticulture Research Institute (Tekirdağ, Turkey); ** YAHCRI, Yalova Atatürk Horticultural Central Research Institutes (Yalova, Turkey).

**Table 2:** Scoring scale used for downy and powdery mildew diseases in grape leaves (from 1: very low, to 9: very high).

Scale	Level	Symptom/Reaction		
		Powdery Mildew	Downy Mildew	Host Response
1	Very low	Tiny spots or no symptoms; neither visible sporulation nor mycelium	Tiny necrotic spots or no symptoms; neither sporulation nor mycelium	Extremely Resistant
3	Low	Limited patches < 2 cm diameter; limited sporulation and mycelium; the presence of *Uncinula* is only indicated by a slight curling of the blade	Small patches < 1 cm in diameter; little sporulation or mycelium	Resistant
5	Medium	Patches usually limited with a diameter of 2–5 cm	Little patches 1–2 cm diameter; more or less strong sporulation; irregular formation of mycelium	Tolerant
7	High	Vast patches; some limited; strong sporulation and abundant mycelium	Vast patches; strong sporulation and abundant mycelium; leaf drop later than below	Susceptible
9	Very high	Very vast unlimited patches or totally attached leaf blades; strong sporulation and abundant mycelium	Vast patches or totally attached leaf blades; strong sporulation and dense mycelium; very early leaf drop	Extremely Susceptible

**Table 3:** Scores of grape cultivars/hybrids after artificial inoculation of downy and powdery mildew diseases (1: High Resistance; 3: Resistance; 5: Tolerance; 7: Susceptible; 9: High Susceptible).

Cultivar/Hybrid	Species	Downy Mildew	Powdery Mildew
Isabella	Interspecies (*V. vinifera* × *V. labrusca*)	1	1
Özer Karası		3	3
FX1-1		3	3
FX1-10		5	1
BX1-166		5	5
KXP-10	*V. vinifera*	5	5
86/1		5	7
Güz Gülü		5	5
Italia		5	7

**Table 4:** Contents of major phenolic compounds before and after downy/powdery mildew diseases in grape leaves.

Cultivars/ Hybrids	Catechin * (mg 100 g^−1^)					
	Before Disease		After Downy Mildew		After Powdery Mildew	
	1st Year	2nd Year	1st Year	2nd Year	1st Year	2nd Year
Isabella	0.09 ± 0.02 ^c^ **	15.63 ± 0.07 ^b^	Nd ***	1.60 ± 0.06 ^e^	0.10 ± 0.00 ^c^	1.14 ± 0.03 ^d^
Özer Karası	0.23 ± 0.02 ^b^	54.50 ± 2.40 ^a^	Nd	3.83 ± 0.01 ^a^	Nd	3.67 ± 4.34 ^cd^
Fx1-1	0.21 ± 0.02 ^b^	0.23 ± 0.00 ^f^	0.09 ± 0.02 ^c^	2.38 ± 0.06 ^d^	Nd	1.18 ± 0.08 ^d^
Fx1-10	0.06 ± 0.01 ^c^	5.63 ± 0.02 ^e^	0.25 ± 0.10 ^b^	0.24 ± 0.04 ^g^	0.13 ± 0.00 ^c^	4.83 ± 0.44 ^bc^
BX1-166	0.29 ± 0.02 ^a^	9.12 ± 0.31 ^d^	0.09 ± 0.01 ^c^	3.52 ± 0.06 ^b^	2.62 ± 0.18 ^c^	8.81 ± 0. 24 ^a^
KXP-10	0.21 ± 0.03 ^b^	53.50 ± 0.42 ^a^	0.87 ± 0.10 ^a^	1.49 ± 0.00 ^e^	45.83 ± 4.35 ^a^	3.86 ± 0.06 ^cd^
86/1	Nd	12.77 ± 0.07 ^c^	Nd	0.17 ± 0.00 ^g^	Nd	1.60 ± 0.03 ^cd^
Güzgülü	Nd	8.38 ± 0.06 ^d^	Nd	2.79 ± 0.07 ^c^	Nd	4.12 ± 0.06 ^b–d^
Italia	Nd	0.09 ± 0.00 ^f^	0.05 ± 0.00 ^c^	0.87 ± 0.15 ^f^	13.67 ± 0.29 ^b^	7.27 ± 0.21 ^b^
**Epicatechin (mg 100 g^−1^)**						
Isabella	Nd	0.71 ± 0.04 ^e^	Nd	0.30 ± 0.00 ^i^	42.52 ± 6.04 ^e^	1.24 ± 0.02 ^cd^
Özer Karası	Nd	12.20 ± 0.42 ^b^	Nd	5.43 ± 0.18 ^d^	127.81 ± 14.15 ^c^	Nd
Fx1-1	13.64 ± 3.14 ^c^	17.30 ± 0.31 ^a^	48.70 ± 5.12 ^c^	2.50 ± 0.11 ^e^	Nd	4.18 ± 0.11 ^ab^
Fx1-10	27.83 ± 3.68 ^a^	0.88 ± 0.01 ^e^	Nd	1.01 ± 0.01 ^h^	Nd	1.79 ± 0.00 ^a^
BX1-166	Nd	4.70 ± 0.06 ^d^	Nd	15.37 ± 0.30 ^a^	20.92 ± 1.71 ^f^	0.68 ± 0.01 ^de^
KXP-10	Nd	11.25 ± 1.03 ^c^	86.66 ± 9.19 ^a^	7.66 ± 0.25 ^b^	101.01 ± 2.41 ^d^	Nd
86/1	Nd	1.17 ± 0.07 ^e^	Nd	1.50 ± 0.07 ^g^	427.00 ± 8.49 ^a^	4.55 ± 5.24 ^a^
Güzgülü	7.58 ± 1.17 ^d^	1.14 ± 0.05 ^e^	63.09 ± 1.66 ^b^	6.40 ± 0.11 ^c^	7.53 ± 0.20 ^fg^	Nd
Italia	18.69 ± 0.69 ^b^	0.62 ± 0.01 ^e^	70.29 ± 4.84 ^b^	1.94 ± 0.02 ^f^	176.27 ± 1.66 ^b^	0.14 ± 0.00 ^e^
**Gallic Acid (mg 100 g^−1^)**						
Isabella	1.24 ± 0.10 ^e^	2.33 ± 0.04 ^c^	0.51 ± 0.07 ^fg^	2.02 ± 0.03 ^a^	2.68 ± 0. 43 ^cd^	3.52 ± 0.03 ^e^
Özer Karası	1.82 ± 0.11 ^a^	2.81 ± 0.01 ^b^	0.28 ± 0.01 ^g^	0.24 ± 0.06 ^g^	2.57 ± 0.29 ^cd^	3.26 ± 0.08 ^f^
Fx1-1	1.56 ± 0.13 ^bc^	1.28 ± 0.11 ^g^	2.53 ± 0.35 ^a^	1.05 ± 0.07 ^c^	1.51 ± 0.03 ^d^	2.61 ± 0.01 ^h^
Fx1-10	1.50 ± 0.09 ^cd^	1.44 ± 0.06 ^f^	0.82 ± 0.09 ^c–e^	0.69 ± 0.13 ^e^	1.86 ± 0.19 ^cd^	2.77 ± 0.14 ^g^
BX1-166	1.31 ± 0.09 ^de^	3.55 ± 0.07 ^a^	0.92 ± 0.02 ^cd^	0.90 ± 0.14 ^d^	1.91 ± 0.20 ^cd^	2.48 ± 0.11 ^i^
KXP-10	0.96 ± 0.09 ^f^	0.55 ± 0.07 ^h^	0.97 ± 0.01 ^c^	0.88 ± 0.11 ^d^	4.91 ± 0.04 ^ab^	4.98 ± 0.11 ^b^
86/1	1.79 ± 0.02 ^ab^	3.62 ± 0.03 ^a^	0.64 ± 0.04 ^d–f^	0.65 ± 0.07 ^e^	4.20 ± 0.36 ^ab^	6.51 ± 0.01 ^a^
Güzgülü	1.63 ± 0.12 ^a–c^	1.86 ± 0.08 ^d^	1.50 ± 0.03 ^b^	1.39 ± 0.13^b^	4.06 ± 1.58 ^ab^	4.53 ± 0.04 ^d^
Italia	1.52 ± 0.07 ^cd^	1.58 ± 0.04 ^e^	0.61 ± 0.05 ^ef^	0.51 ± 0.02 ^f^	3.02 ± 0.09 ^bc^	4.78 ± 0.11 ^c^

* For each phenolic compound within a column, different superscripts across the table indicate significant differences at *p* ≤ 0.05. ** All means are expressed as means ± standard deviation (*n* = 3). *** Nd = not detected.

**Table 5:** Pearson’s correlation coefficients (R) among major phenolic compounds before (BD) and after downy mildew (DM) or powdery mildew diseases (PM) in grape leaves (*n* = 9).

Variable, Disease Condition	By Variable, Disease Condition	Correlation Coefficient	Lower 95%	Upper 95%	Significance Probability	Sign of Correlation
Gallic acid, DM	Gallic acid, BD	−0.3561	−0.8251	0.4034	0.3468	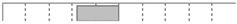
Gallic acid, PM	Gallic acid, BD	−0.0298	−0.6805	0.6471	0.9393	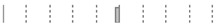
	Gallic acid, DM	−0.2248	−0.7735	0.5164	0.5608	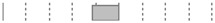
Catechin, BD	Gallic acid, BD	−0.1289	−0.7305	0.5853	0.7410	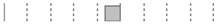
	Gallic acid, DM	−0.4373	−0.8535	0.3197	0.2392	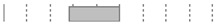
	Gallic acid, PM	0.2791	−0.4726	0.7957	0.4671	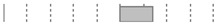
Catechin, DM	Gallic acid, BD	0.0781	−0.6181	0.7056	0.8416	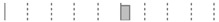
	Gallic acid, DM	0.1181	−0.5925	0.7253	0.7621	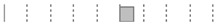
	Gallic acid, PM	−0.3554	−0.8248	0.4041	0.3480	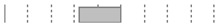
	Catechin, BD	0.4408	−0.3158	0.8547	0.2351	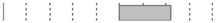
Catechin, PM	Gallic acid, BD	−0.6395	−0.9150	0.0428	0.0637	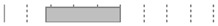
	Gallic acid, DM	−0.1769	−0.7526	0.5521	0.6488	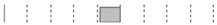
	Gallic acid, PM	0.4257	−0.3323	0.8496	0.2532	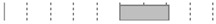
	Catechin, BD	0.4882	−0.2603	0.8702	0.1824	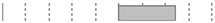
	Catechin, DM	0.0616	−0.6282	0.6972	0.8749	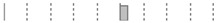
Epicatechin, BD	Gallic acid, BD	−0.5155	−0.8788	0.2259	0.1554	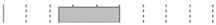
	Gallic acid, DM	0.1934	−0.5401	0.7599	0.6180	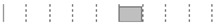
	Gallic acid, PM	−0.4900	−0.8708	0.2581	0.1806	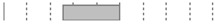
	Catechin, BD	−0.2703	−0.7922	0.4800	0.4818	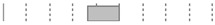
	Catechin, DM	−0.1225	−0.7274	0.5896	0.7536	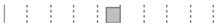
	Catechin, PM	−0.0092	−0.6692	0.6589	0.9812	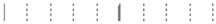
Epicatechin, DM	Gallic acid, BD	−0.6992	−0.9310	−0.0654	0.0361 *	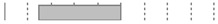
	Gallic acid, DM	0.2901	−0.4632	0.8001	0.4488	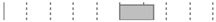
	Gallic acid, PM	0.3828	−0.3772	0.8347	0.3092	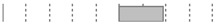
	Gallic acid, BD	0.0931	−0.6087	0.7131	0.8118	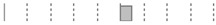
	Catechin, DM	0.1435	−0.5755	0.7373	0.7127	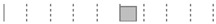
	Catechin, PM	0.7056	0.0781	0.9327	0.0337*	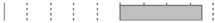
	Epicatechin, BD	0.2261	−0.5154	0.7740	0.5585	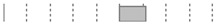
Epicatechin, PM	Gallic acid, BD	0.4776	−0.2732	0.8668	0.1935	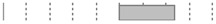
	Gallic acid, DM	−0.5198	−0.8801	0.2204	0.1515	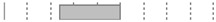
	Gallic acid, PM	0.6913	0.0502	0.9289	0.0392 *	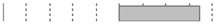
	Catechin, BD	0.1081	−0.5990	0.7205	0.7819	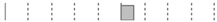
	Catechin, DM	−0.4882	−0.8702	0.2604	0.1825	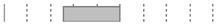
	Catechin, PM	0.0093	−0.6589	0.6693	0.9811	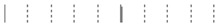
	Epicatechin, BD	−0.4049	−0.8425	0.3545	0.2797	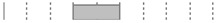
	Epicatechin, DM	−0.1681	−0.7486	0.5584	0.6655	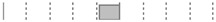

* indicates that the correlation coefficient is statistically significant at *p* ≤ 0.05.

**Table 6:** Catechin contents (mg 100 g^−1^) of pulps, skins, and seeds of grape cultivars/hybrids used in the study in two growing seasons.

Cultivar/ Hybrid	Pulp *		Skin		Seed	
	1st Year	2nd Year	1st Year	2nd Year	1st Year	2nd Year
Isabella	0.81 ± 0.03 ^a^ **	0.76 ± 0.01 ^a^	1.86 ± 0.07 ^e^	1.88 ± 0.01 ^d^	48.17 ± 2.99 ^m^	44.95 ± 1.55 ^l^
Alphonse L.	0.70 ± 0.03 ^cd^	0.68 ± 0.02 ^b–d^	3.07 ± 0.05 ^c^	3.02 ± 0.22 ^ab^	103.18 ± 2.70 ^k^	100.80 ± 5.60 ^ij^
Muscat Ham.	0.72 ± 0.01 ^bc^	0.69 ± 0.02 ^bc^	1.21 ± 0.02 ^h^	1.20 ± 0.10 ^f^	176.05 ± 2.85 ^f^	176.55 ± 2.25 ^ef^
Yalova Misketi	0.69 ± 0.11^c–e^	0.65 ± 0.00 ^d–g^	0.69 ± 0.51 ^j^	0.11 ± 0.01 ^j^	262.11 ± 6.20 ^c^	263.60 ± 3.20 ^c^
Trakya İlkeren	0.65 ± 0.02 ^c–g^	0.68 ± 0.01 ^b–d^	3.28 ± 0.06 ^b^	3.22 ± 0.36 ^a^	187.45 ± 1.96 ^e^	187.10 ± 10.80 ^de^
Bilecik İrikarası	0.69 ± 0.06 ^c–e^	0.66 ± 0.02 ^c–f^	0.76 ± 0.02 ^j^	0.74 ± 0.06 ^h^	117.85 ± 1.84 ^j^	118.00 ± 3.90 ^i^
İsmetbey	0.63 ± 0.02 ^d–g^	0.64 ± 0.01 ^e–g^	0.04 ± 0.00 ^l^	0.04 ± 0.00 ^j^	130.86 ± 1.73 ^i^	135.00 ± 3.20 ^h^
KXP-10	0.68 ± 0.06 ^c–f^	0.69 ± 0.00 ^bc^	2.55 ± 0.06 ^d^	2.49 ± 0.44 ^c^	491.91 ± 2.76 ^a^	494.95 ± 18.05 ^a^
Özer Karası	0.66 ± 0.08 ^c–g^	0.65 ± 0.04 ^d–g^	1.73 ± 0.05 ^ef^	1.73 ± 0.01 ^de^	163.73 ± 2.02 ^g^	163.50 ± 17.80 ^fg^
Tekirdağ Çekirdeksizi	0.69 ± 0.05 ^c–e^	0.65 ± 0.06 ^d–g^	0.48 ± 0.04 ^k^	0.44 ± 0.02 ^i^	Seedless	Seedless
Reçel Üzümü	0.79 ± 0.08 ^ab^	0.71 ± 0.02 ^b^	0.47 ± 0.04 ^k^	0.44 ± 0.01 ^i^	Seedless	Seedless
Güz Gülü	0.60 ± 0.02 ^f–h^	0.66 ± 0.01 ^d–g^	Nd ***	Nd	Seedless	Seedless
Pembe 77	0.62 ± 0.03 ^d–g^	0.66 ± 0.01 ^d–g^	1.67 ± 0.06 ^fg^	1.60 ± 0.03 ^e^	151.56 ± 6.80 ^h^	154.10 ± 19.70 ^g^
Uslu	0.60 ± 0.05 ^f–h^	0.66 ± 0.01 ^d–g^	2.58 ± 0.04 ^d^	2.61 ± 0.05 ^c^	191.78 ± 1.17 ^de^	191.93 ± 0.88 ^d^
83/1	0.65 ± 0.05 ^c–g^	0.67 ± 0.01 ^c–e^	0.77 ± 0.02 ^j^	0.77 ± 0.11 ^gh^	197.16 ± 1.23 ^d^	198.35 ± 1.85 ^d^
FX1-1	0.52 ± 0.04 ^hi^	0.58 ± 0.02 ^h^	1.15 ± 0.07 ^hi^	1.10 ± 0.05 ^f^	83.42 ± 2.97 ^l^	82.65 ± 2.04 ^k^
85/1	0.66 ± 0.03 ^c–g^	0.67 ± 0.02 ^c–e^	4.59 ± 0.05 ^a^	2.87 ± 0.06 ^b^	370.58 ± 7.06 ^b^	367.70 ± 2.50 ^b^
BX1-166	0.50 ± 0.03 ^i^	0.54 ± 0.05 ^i^	1.00 ± 0.02 ^i^	0.98 ± 0.03 ^fg^	113.59 ± 1.78 ^j^	109.00 ± 6.86 ^ij^
53/1	0.61 ± 0.02 ^e–g^	0.67 ± 0.02 ^cd^	0.03 ± 0.01 ^l^	0.03 ± 0.00 ^j^	117.26 ± 2.67 ^j^	171.80 ± 3.60 ^f^
86/1	0.68 ± 0.06 ^c–f^	0.63 ± 0.00 ^fg^	1.53 ± 0.10 ^g^	3.20 ± 0.10 ^a^	170.89 ± 1.46 ^f^	170.25 ± 2.95 ^f^
FX1-10	0.59 ± 0.03 ^gh^	0.63 ± 0.02 ^g^	1.08 ± 0.05 ^hi^	1.12 ± 0.10 ^f^	100.84 ± 0.91 ^k^	101.33 ± 2.19 ^j^
130/1	0.67 ± 0.02 ^c–g^	0.70 ± 0.02 ^bc^	Nd	Nd	Seedless	Seedless

* Different superscripts within a column indicate significant differences at *p* ≤ 0.05. ** All means are expressed as means ± standard deviations (*n* = 3). *** Nd = not detected.

**Table 7:** Epicatechin contents (mg 100 g^−1^) of pulps, skins, and seeds of grape cultivars/hybrids used in the study in two growing seasons.

Cultivar/ Hybrid	Pulp *		Skin		Seed	
	1st Year	2nd Year	1st Year	2nd Year	1st Year	2nd Year
Isabella	0.05 ± 0.00 ^e–h^ **	Nd ***	Nd	Nd	20.65 ± 0.17 ^n^	20.70 ± 0.90 ^j^
Alphonse L.	0.24 ± 0.01 ^b–d^	0.23 ± 0.02 ^d^	1.22 ± 0.01 ^d^	1.21 ± 0.03 ^c^	44.72 ± 0.87 ^jk^	44.65 ± 1.4 ^h^
Muscat Ham.	0.12 ± 0.01 ^e–g^	0.12 ± 0.01 ^fg^	0.28 ± 0.03 ^j^	0.26 ± 0.04 ^f^	52.48 ± 0.71 ^i^	51.70 ± 0.70 ^g^
Yalova Misketi	0.01 ± 0.00 ^gh^	0.01 ± 0.00 ^i^	Nd	Nd	72.74 ± 0.81 ^d^	73.50 ± 1.10 ^d^
Trakya İlkeren	0.16 ± 0.00 ^c–e^	0.16 ± 0.02 ^ef^	1.39 ± 0.02 ^b^	1.37 ± 0.01 ^b^	56.13 ± 3.24 ^h^	58.60 ± 1.20 ^f^
Bilecik İ.K.	0.02 ± 0.00 ^gh^	0.01 ± 0.00 ^i^	1.79 ± 0.02 ^a^	1.79 ± 0.08 ^a^	43.01 ± 1.32 ^kl^	42.50 ± 3.70 ^h^
İsmetbey	1.06 ± 0.03 ^a^	1.03 ± 0.07 ^b^	Nd	Nd	33.88 ± 1.62 ^m^	35.65 ± 0.85 ^i^
KXP-10	0.03 ± 0.00 ^f–h^	0.91 ± 0.01 ^c^	0.91 ± 0.01 ^f^	0.91 ± 0.01 ^d^	99.45 ± 1.91 ^b^	99.65 ± 0.55 ^b^
Özer Karası	0.29 ± 0.02 ^b^	Nd	Nd	Nd	20.95 ± 0.81 ^n^	20.50 ± 2.30 ^j^
Tekirdağ Ç.	0.14 ± 0.02 ^d–f^	0.14 ± 0.02 ^ef^	0.10 ± 0.00 ^m^	0.10 ± 0.02 ^g^	Seedless	Seedless
Reçel Üzümü	0.02 ± 0.00 ^gh^	0.01 ± 0.00 ^i^	Nd	Nd	Seedless	Seedless
Güz Gülü	Nd	Nd	Nd	Nd	Seedless	Seedless
Pembe 77	0.02 ± 0.00 ^gh^	1.23 ± 0.11 ^a^	1.25 ± 0.02 ^c^	1.23 ± 0.11 ^c^	79.52 ± 0.46 ^c^	79.30 ± 9.22 ^c^
Uslu	Nd	Nd	Nd	Nd	66.44 ± 2.17 ^e^	66.80 ± 1.90 ^e^
83/1	Nd	Nd	0.67 ± 0.02 ^g^	0.65 ± 0.00 ^e^	46.17 ± 2.59 ^j^	45.85 ± 0.35 ^h^
FX1-1	0.26 ± 0.18 ^bc^	0.24 ± 0.06 ^d^	0.22 ± 0.03 ^k^	Nd	62.46 ± 1.70 ^g^	59.25 ± 3.53 ^f^
85/1	0.23 ± 0.01 ^b–d^	0.23 ± 0.06 ^d^	1.09 ± 0.03 ^e^	Nd	63.42 ± 0.50 ^fg^	62.90 ± 0.30 ^ef^
BX1-166	0.08 ± 0.01 ^e–h^	0.06 ± 0.02 ^hi^	0.32 ± 0.03 ^i^	Nd	36.11 ± 0.56 ^m^	34.14 ± 1.92 ^i^
53/1	0.31 ± 0.22 ^b^	0.19 ± 0.01 ^de^	Nd	Nd	40.73 ± 1.15 ^l^	45.40 ± 3.90 ^h^
86/1	Nd	Nd	0.56 ± 0.03 ^h^	Nd	106.91 ± 1.15 ^a^	107.20 ± 6.10 ^a^
FX1-10	0.11 ± 0.08 ^e–g^	0.08 ± 0.04 ^gh^	0.14 ± 0.02 ^l^	0.11 ± 0.03 ^g^	65.80 ± 1.42 ^ef^	61.15 ± 2.02 ^f^
130/1	Nd	Nd	Nd	Nd	Seedless	Seedless

* Different superscripts within a column indicate significant differences at *p* ≤ 0.05. ** All means are expressed as means ± standard deviations (*n* = 3). *** Nd = not detected.

**Table 8:** Gallic acid contents of pulps, skins, and seeds of grape cultivars/hybrids used in the study in two growing seasons (mg 100 g^−1^).

Cultivar/ Hybrid	Pulp *		Skin		Seed	
	1st Year	2nd Year	1st Year	2nd Year	1st Year	2nd Year
Isabella	0.76 ± 0.01 ^a^ **	0.76 ± 0.01 ^a^	0.59 ± 0.03 ^d^	0.59 ± 0.01 ^k^	1.35 ± 0.10 ^i^	1.26 ± 0.05 ^k^
Alphonse L.	0.69 ± 0.02 ^c–e^	0.67 ± 0.01 ^c–e^	0.85 ± 0.03 ^c^	0.82 ± 0.01 ^d^	4.50 ± 0.31 ^d^	4.16 ± 0.20 ^ef^
Muscat Ham.	0.68 ± 0.01 ^c–f^	0.69 ± 0.00 ^c^	0.72 ± 0.01 ^cd^	0.72 ± 0.01 ^ef^	3.93 ± 0.10 ^e^	3.95 ± 0.05 ^fg^
Yalova Misketi	0.65 ± 0.01 ^h–j^	0.65 ± 0.00 ^fg^	1.20 ± 0.18 ^b^	0.98 ± 0.05 ^c^	3.71 ± 0.06 ^e^	3.72 ± 0.03 ^g^
Trakya İlkeren	0.68 ± 0.03 ^c–f^	0.68 ± 0.01 ^cd^	0.68 ± 0.03 ^cd^	0.67 ± 0.00 ^f–i^	5.27 ± 0.64 ^c^	5.47 ± 0.27 ^b^
Bilecik İ.K.	0.66 ± 0.01 ^g–i^	0.66 ± 0.01 ^d–f^	0.63 ± 0.02 ^d^	0.63 ± 0.02 ^h–k^	1.58 ± 0.13 ^i^	1.47 ± 0.05 ^k^
İsmetbey	0.64 ± 0.02 ^i–j^	0.64 ± 0.01 ^gh^	0.69 ± 0.04 ^cd^	0.69 ± 0.00 ^fg^	2.17 ± 0.10 ^h^	4.73 ± 0.08 ^d^
KXP-10	0.70 ± 0.02 ^bc^	0.69 ± 0.00 ^bc^	0.75 ± 0.03 ^cd^	0.76 ± 0.07 ^e^	6.88 ± 0.04 ^a^	6.88 ± 0.22 ^a^
Özer Karası	0.65 ± 0.02 ^h–j^	0.65 ± 0.04 ^c–g^	0.65 ± 0.04 ^d^	0.65 ± 0.01 ^g–j^	3.08 ± 0.04 ^f^	3.04 ± 0.26 ^hi^
Tekirdağ Ç.	0.64 ± 0.01 ^i–j^	0.65 ± 0.06 ^fg^	0.59 ± 0.02 ^d^	0.59 ± 0.02 ^k^	Seedless	Seedless
Reçel Üzümü	0.72 ± 0.00 ^b^	0.72 ± 0.01 ^b^	0.63 ± 0.01 ^d^	0.62 ± 0.00 ^i–k^	Seedless	Seedless
Güz Gülü	0.66 ± 0.01 ^g–i^	0.66 ± 0.01 ^d–f^	0.64 ± 0.01 ^d^	0.63 ± 0.01 ^h–k^	Seedless	Seedless
Pembe 77	0.66 ± 0.01 ^g–i^	0.66 ± 0.01 ^d–f^	1.32 ± 0.03 ^ab^	1.31 ± 0.05 ^b^	2.92 ± 0.06 ^fg^	2.98 ± 0.31 ^hi^
Uslu	0.66 ± 0.02 ^g–i^	0.66 ± 0.01 ^d–f^	0.73 ± 0.03 ^cd^	0.72 ± 0.01 ^ef^	2.67 ± 0.15 ^g^	2.54 ± 0.03 ^j^
83/1	0.68 ± 0.03 ^c–f^	0.67 ± 0.01 ^c–e^	1.47 ± 0.07 ^a^	1.43 ± 0.11 ^a^	3.21 ± 0.02 ^f^	3.21 ± 0.02 ^h^
FX1-1	0.57 ± 0.03 ^l^	0.56 ± 0.03 ^i^	0.61 ± 0.02 ^d^	0.61 ± 0.01 ^jk^	4.40 ± 0.38 ^d^	4.25 ± 0.27 ^e^
85/1	0.67 ± 0.02 ^e–h^	0.67 ± 0.02 ^c–e^	1.42 ± 0.49 ^a^	0.68 ± 0.01 ^f–h^	3.86 ± 0.18 ^e^	3.93 ± 0.02 ^fg^
BX1-166	0.59 ± 0.02 ^l^	0.60 ± 0.01 ^h^	0.59 ± 0.03 ^d^	0.58 ± 0.04 ^k^	2.88 ± 0.05 ^fg^	2.90 ± 0.08 ^i^
53/1	0.68 ± 0.01 ^c–f^	0.68 ± 0.00 ^cd^	0.63 ± 0.01 ^d^	0.62 ± 0.02 ^i–k^	2.27 ± 0.10 ^h^	5.01 ± 0.09 ^c^
86/1	0.63 ± 0.02 ^jk^	0.63 ± 0.00 ^gh^	1.44 ± 0.11 ^a^	0.82 ± 0.01 ^d^	3.74 ± 0.05 ^e^	3.77 ± 0.04 ^g^
FX1-10	0.62 ± 0.01 ^k^	0.63 ± 0.01 ^gh^	0.64 ± 0.03 ^d^	0.66 ± 0.02 ^g–j^	6.35 ± 0.44 ^b^	6.63 ± 0.11 ^ab^
130/1	0.70 ± 0.02 ^bc^	0.69 ± 0.00 ^c^	0.68 ± 0.01 ^cd^	0.65 ± 0.00 ^g–j^	Seedless	Seedless

* Different superscripts within a column indicate significant differences at *p* ≤ 0.05. ** All means are expressed as means ± standard deviation (*n* = 3).

**Table 9:** Pearson’s correlation coefficients (R) among major phenolic compounds in different parts of grapes (seeds, skins, and pulps) (*n* = 22).

Variable (Compound, Part)	By Variable (Compound, Part)	Correlation Coefficient	Lower 95%	Upper 95%	Significance Probability	Sign of Correlation
Gallic acid (Skin)	Gallic acid (Pulp)	−0.0155	−0.4343	0.4088	0.9454	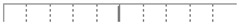
Catechin (Pulp)	Gallic acid (Pulp)	0.0866	−0.3477	0.4903	0.7015	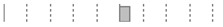
	Gallic acid (Skin)	0.9538	0.8901	0.9809	<0.0001 *	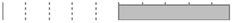
Catechin (Skin)	Gallic acid (Pulp)	0.1320	−0.3067	0.5244	0.5583	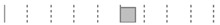
	Gallic acid (Skin)	0.2235	−0.2187	0.5896	0.3173	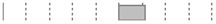
	Catechin (Pulp)	0.3376	−0.0980	0.6646	0.1244	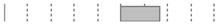
Epicatechin (Pulp)	Gallic acid (Pulp)	−0.1293	−0.5224	0.3092	0.5664	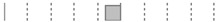
	Gallic acid (Skin)	0.0713	−0.3611	0.4786	0.7524	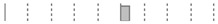
	Catechin (Pulp)	−0.0011	−0.4225	0.4207	0.9960	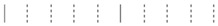
	Catechin (Skin)	0.0003	−0.4213	0.4219	0.9988	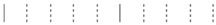
Epicatechin (Skin)	Gallic acid (Pulp)	0.1002	−0.3356	0.5007	0.6572	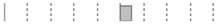
	Gallic acid (Skin)	0.2852	−0.1551	0.6309	0.1983	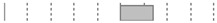
	Catechin (Pulp)	0.2420	−0.2000	0.6022	0.2778	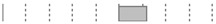
	Catechin (Skin)	0.4435	0.0269	0.7288	0.0387 *	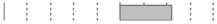
	Epicatechin (Pulp)	0.1315	−0.3071	0.5241	0.5597	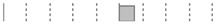
Gallic acid (Seed)	Gallic acid (Pulp)	−0.2728	−0.6228	0.1682	0.2194	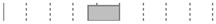
	Gallic acid (Skin)	0.2028	−0.2393	0.5752	0.3655	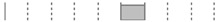
	Catechin (Pulp)	0.1714	−0.2697	0.5531	0.4456	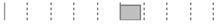
	Catechin (Skin)	0.5039	0.1045	0.7633	0.0168 *	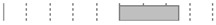
	Epicatechin (Pulp)	0.3185	−0.1191	0.6525	0.1486	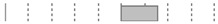
	Epicatechin (Skin)	0.2882	−0.1519	0.6329	0.1934	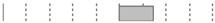
Epicatechin (Seed)	Gallic acid (Pulp)	−0.2651	−0.6176	0.1762	0.2332	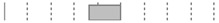
	Gallic acid (Skin)	0.5137	0.1175	0.7688	0.0145 *	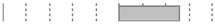
	Catechin (Pulp)	0.5203	0.1264	0.7724	0.0131 *	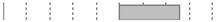
	Catechin (Skin)	0.5358	0.1475	0.7810	0.0102 *	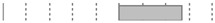
	Epicatechin (Pulp)	0.2019	−0.2402	0.5746	0.3677	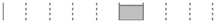
	Epicatechin (Skin)	0.3505	−0.0834	0.6727	0.1098	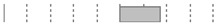
	Gallic acid (Seed)	0.7746	0.5243	0.9017	<0.0001 *	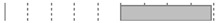
Catechin (Seed)	Gallic acid (Pulp)	0.0129	−0.4109	0.4322	0.9545	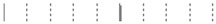
	Gallic acid (Skin)	0.4117	−0.0120	0.7101	0.0570	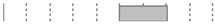
	Catechin (Pulp)	0.4531	0.0389	0.7344	0.0342 *	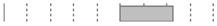
	Catechin (Skin)	0.5494	0.1664	0.7884	0.0081 *	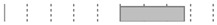
	Epicatechin (Pulp)	0.2565	−0.1851	0.6120	0.2491	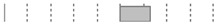
	Epicatechin (Skin)	0.3047	−0.1342	0.6436	0.1680	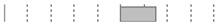
	Gallic acid (Seed)	0.6983	0.3921	0.8652	0.0003 *	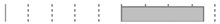
	Epicatechin (Seed)	0.7388	0.4607	0.8848	<0.0001 *	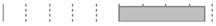

* indicates that the correlation coefficient is statistically significant at *p* ≤ 0.05.

## Data Availability

The authors will make the results available if requested.
